# Live Quantitative Monitoring of Mineral Deposition in Stem Cells Using Tetracycline Hydrochloride

**DOI:** 10.1089/ten.tec.2017.0400

**Published:** 2018-03-01

**Authors:** Laura Macri-Pellizzeri, Nigel De Melo, Ifty Ahmed, David Grant, Brigitte Scammell, Virginie Sottile

**Affiliations:** ^1^ Wolfson STEM Centre, Division of Cancer and Stem Cells, School of Medicine, The University of Nottingham, Nottingham, United Kingdom.; ^2^ Advanced Materials Group, Department of Mechanical, Materials and Manufacturing Engineering, Faculty of Engineering, The University of Nottingham, Nottingham, United Kingdom.; ^3^ Orthopaedics and Trauma Group, Division of Rheumatology, Orthopaedics, and Dermatology, School of Medicine, The University of Nottingham, Nottingham, United Kingdom.

**Keywords:** stem cells, osteogenesis, *in vitro* differentiation, live cell imaging, quantitative assay

## Abstract

The final stage of *in vitro* osteogenic differentiation is characterized by the production of mineral deposits containing calcium cations and inorganic phosphates, which populate the extracellular matrix (ECM) surrounding the cell monolayer. Conventional histological techniques for the assessment of mineralization, such as Von Kossa and Alizarin Red S staining, are end point techniques requiring cell fixation. Moreover, in both cases staining quantitation requires dye extraction, which irreversibly alters the ECM conformation and structure, therefore preventing the use of the sample for further analysis. In this study, the use of tetracycline hydrochloride (TC) is proposed for the nondestructive staining, quantitation, and imaging of mineralizing bone-like nodules in live cultures of human bone marrow mesenchymal stem cells cultured under osteogenic conditions. Overnight administration of TC to living cells was shown not to alter the metabolic activity or the progression of cell differentiation. When applied to differentiating cultures, cell exposure to serial doses of TC was found to produce quantifiable fluorescence emission specifically in osteogenic cultures. Incubation with TC enabled fluorescence imaging of mineralized areas in live cultures and the combination with other fluorophores using appropriate filters. These results demonstrate that serial TC administration over the differentiation time course provides a qualitative and quantitative tool for the monitoring and evaluation of the differentiation process in live cells.

## Introduction

The cellular process underpinning bone formation is routinely modeled *in vitro* using different types of cells, including primary osteoblasts and multipotent and pluripotent stem cells which, under specific physicochemical stimulation, differentiate into mineralizing bone-like cells.^1^ During osteogenic differentiation, a well-organized and collagen-enriched extracellular matrix (ECM) is formed followed by the production of extracellular mineral deposits made of calcium and inorganic phosphates.^2^

In conventional 2D culture systems, these mineral deposits can be identified on top of the cell monolayer through brightfield imaging.^3^ However, qualitative and quantitative assessments of mineralization are essential for the full characterization of differentiation and are conventionally achieved with histological stain techniques. The most widely used assays include Von Kossa and Alizarin Red S staining, which respectively target anionic phosphates and calcium cations.^4,5^ However, both methods are end point assays and require cell fixation, followed by multiple staining steps. To overcome these limitations and enable live analyses, diverse fluorochrome-based labeling methods have been proposed for the evaluation of bone formation *in vitro* and *in vivo* such as Giemsa, Calcein blue, and Xylenol Orange, among others.^6–9^

Tetracyclines (TCs) constitute a wide family of broad spectrum antibiotics classified as natural, semisynthetic, and chemically modified according to their origin.^10^ In addition to their antimicrobial activity, these compounds are characterized by their calcium chelating ability and fluorescence emission.^11,12^ These properties have led TCs to be used as a marker of calcification front in bone, applied *in vivo* by parenteral or enteral administration or used for staining bone biopsies postfixation.^13^ TCs have also more recently been used for the qualitative observation of mineralized ECM in dental pulp cell culture by fluorescence imaging.^14^ However, TCs have not yet been used for the quantitative evaluation of mineralization in live cultures.

The aim of this study was to investigate the use of tetracycline hydrochloride for the nondestructive *in vitro* staining, quantification, and live imaging of bone-like mineralized ECM using differentiating human mesenchymal stem cells (MSCs).

## Materials and Methods

All reagents were purchased from ThermoFisher Scientific (UK) unless otherwise stated.

### Cell culture and differentiation

Immortalized human bone marrow-derived MSCs^15–18^ were seeded at a density of 4000 cells/cm^2^ in 48-well plates. After 24 h, the standard medium (SC) (low-glucose Dulbecco's modified Eagle's medium supplemented with 10% fetal calf serum, 1% penicillin and streptomycin, 1% L-Glutamine, and 1% of nonessential amino acids) was replaced with osteogenic medium (OS) (SC supplemented with 0.1 μM dexamethasone, 10 mM β-glycerophosphate, and 50 μM ascorbic acid; Sigma-Aldrich, UK) to induce osteogenic lineage differentiation.^19^ Cells were cultured for 21 days at 37°C and 5% CO_2_, with medium refreshment every 48 h.

### Evaluation of cytotoxicity

Cell metabolic activity was analyzed using PrestoBlue reagent at days 7, 14, and 21, according to the manufacturer's instructions. Briefly, the cells were washed once with phosphate buffered saline (PBS) and incubated with 300 μL of SC containing 10% of PrestoBlue reagent at 37°C for 40 min which was within the dynamic range of the assay. Two hundred fifty microliters were transferred to a new 96-well plate, and the fluorescence was measured in a microplate reader (Tecan Infinite 200) using excitation and emission wavelengths set at 560 and 590 nm, respectively.

### Alkaline phosphatase assay and Alizarin Red S staining

Alkaline phosphatase (ALP) activity was assayed at days 7 and 14 of culture. A solution containing 1 mg/mL p-nitrophenyl phosphate and 0.2 M Tris buffer (SIGMAFAST; Sigma-Aldrich) was prepared according to the manufacturer's instructions. Cells seeded in 48-well plates were washed twice with PBS, and 300 μL of assay solution was added to each well. ALP activity was monitored by performing 12 readings of the optical density at 405 nm over 24 min in a microplate reader. Cells were then washed twice, and fresh medium was added before returning the cells to the incubator until the following time point.

Alizarin Red S staining was performed at days 7, 14, or 21 as stated. Cell fixation was performed using 4% paraformaldehyde for 10 min at 4°C. Before the staining, fixed cells were washed twice with deionized water. Then, 200 μL of 1% w/v Alizarin Red S solution (Sigma-Aldrich) was added to each well for 10–15 min, followed by extensive washing with deionized water before imaging. For Alizarin Red S staining quantification, stained cells were washed with deionized water and incubated with 200 μL of destaining solution (20% methanol, 10% acetic acid in deionized water) during 15–20 min before measuring the absorbance of the solution at 405 nm in the microplate reader.

### Tetracycline administration and analysis of fluorescence emission

The tetracycline staining solution chosen was prepared with tetracycline hydrochloride (TC) (Sigma-Aldrich) dissolved in PBS, filtered through 0.22 μm syringe filter, and administrated to the cells at final concentrations of 5, 10, 20, or 40 μg/mL in culture medium at days 6, 13, and 20. After overnight incubation (18–22 h) with TC, cells were washed twice with PBS; then, 300 μL of PBS was added to each well, and fluorescence was measured using the microplate reader (Tecan Infinite 200), recording 25 reading points per well at 390 nm excitation and 560 nm emission.

### Cell staining and imaging

For both live and fixed samples, cell nuclei were counterstained by incubating cells with 10 μg/mL of Hoechst 33258 for 10 min. Cytoskeletal actin fibers were visualized in fixed cells using VECTASHIELD mounting medium containing TRITC-Phalloidin (Vector Laboratories, UK).

Live cells were imaged on a Leica DM IRB microscope using the A filter cube (excitation filter λ 340–380 nm, emission long pass filter λ 425 nm) and coupled to a QICAM Fast 1394 camera. Confocal laser scanning microscopy was carried out on a ZEISS Elyra PS.1 microscope equipped with LSM 780 confocal unit using 10 × /0.45NA water immersion objective. The TC emission spectrum was acquired in Lambda mode using a 32-channel meta-detector in the confocal microscope. After inspecting the emission signals, sequential imaging channels were set for each fluorophore, and for each channel the respective laser was assigned: Hoechst: λ_ex._ 405 nm laser, λ_em._ 405–437 nm; TC: λ_ex._ 405 nm laser, λ_em._ 588–650 nm; and TRITC-Phalloidin: λ_ex._ 561 nm laser, λ_em._ 579–641 nm.

### Statistics

Results from three independent experiments are presented as mean ± standard error of the mean. One-way analysis of variance with Tukey's multiple comparison *post hoc* test was used. A 95% confidence level was considered significant. Statistical analysis was performed with the GraphPad PRISM 7.01 software package.

## Results

### Tetracycline as an *in vitro* live stain for osteogenic differentiation

**FIG. 1.**
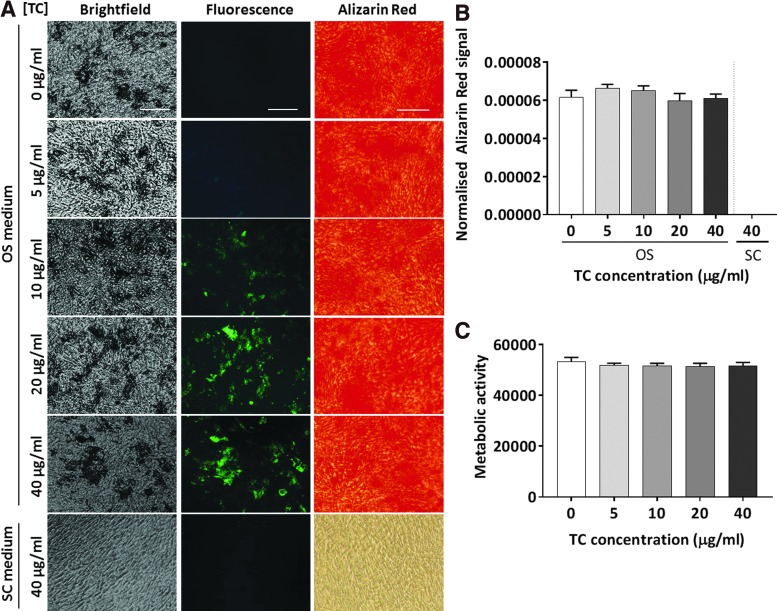
Human MSC cultures exposed to increasing doses of TC at day 20 of differentiation treatment. Scale bar: 125 μm. **(A)** Representative images of cells cultured in SC or OS media after TC (*green*) and Alizarin Red S staining. **(B, C)** Quantification of Alizarin Red S staining **(B)** and metabolic activity **(C)**. (*n* = 3). MSCs, mesenchymal stem cells; OS, osteogenic medium; SC, standard medium; TC, tetracycline. Color images available online at www.liebertpub.com/tec

To test the ability of TC to stain mineralizing cells in culture and determine the optimal working concentration, differentiated and undifferentiated MSCs cultured for 20 days *in vitro* were incubated for 18–22 h with four TC concentrations. Live fluorescence imaging revealed a green staining pattern that colocalized with the grainy mineral deposits visible on the top of the cell monolayer in brightfield mode. The signal was stronger and more defined as the TC concentration increased, while no staining was detected in either differentiated cells unexposed to TC or in undifferentiated cells treated with 40 μg/mL of TC (Fig. 1A).

Alizarin Red S staining performed at day 21 in postfixed cells confirmed the differentiated phenotype of cells transiently exposed to the four concentrations of TC and showed no significant differences between all treatment groups (*p* = 0.47), confirming that TC exposure did not interfere with the staining (Fig. 1A, B). Semiquantitation of DNA amount (Supplementary Fig. S1; Supplementary Data are available online at www.liebertpub.com/tec) and analysis of metabolic activity (Fig. 1C) performed in cells treated with increasing TC concentrations also showed no differences (*p* = 0.77) among the different groups, confirming that the TC treatment was nontoxic at all concentrations tested in this study.

**FIG. 2.**
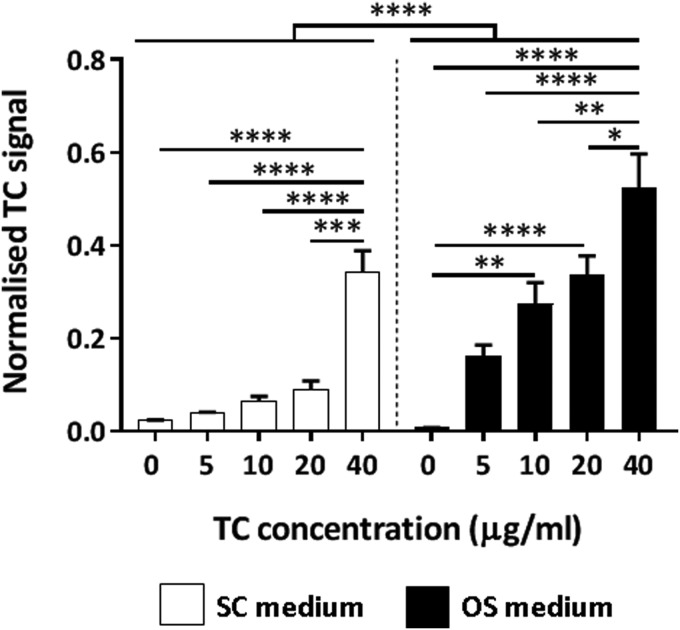
Quantification of TC fluorescence in MSCs cultured during 21 days in SC (*white*) or OS (*black*) medium containing increasing TC doses. SC, standard medium; OS, osteogenic medium. *p < 0.05, **p < 0.01, ***p < 0.001, ****p < 0.0001.

The fluorescence intensity observed in TC-treated cultures was measured using a microplate reader, and TC signal was significantly higher in cells treated with osteogenic condition than in undifferentiated cells maintained in SC medium (Fig. 2). The 40 μg/mL concentration resulted in significantly higher values in comparison to all the other concentrations in both culture conditions (Fig. 2).

### Tetracycline fluorescence spectrum and co-staining with other fluorescent dyes

To define the optimal imaging settings for TC cell labelling, the lambda mode of the confocal microscope was used with 405, 488, 561, or 633 nm lasers to detect the whole TC spectra in fixed cells treated with 20 μg/mL of TC. Strong emission was detected when a 405 nm laser was used with two major peaks at 520 and 584 nm. Importantly, relatively low signals (<8%) were observed in the emission spectra acquired at other wavelengths (488, 561, and 633 nm), suggesting little or no signal spillover (Supplementary Fig. S2).

**FIG. 3.**
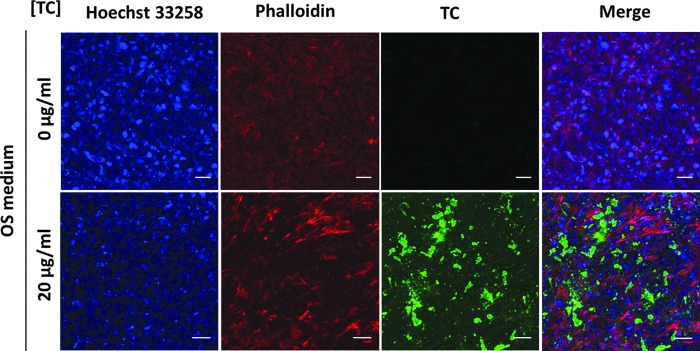
Representative images of OS-treated cells at day 21 stained with TC 20 μg/mL (*green*) or only phosphate buffered saline and counterstained with Hoechst 33258 (*blue*) and Phalloidin (*red*). Scale bar: 125 μm. Color images available online at www.liebertpub.com/tec

To test the possibility of using TC concomitantly with other fluorescent dyes, fixed cells treated with 20 μg/mL of TC were co-stained with Hoechst 33258 and TRITC-Phalloidin to label nuclei and cytoskeleton, respectively. Notably, using the 405 nm laser, a residual Hoechst 33258 signal was detected at 520 nm but not at 584 nm. Therefore, the TC imaging protocol was optimized using the 405 nm laser with detection in the range of 588–650 nm, providing a specific TC signal without any spillover signal from Hoechst 33258 or Phalloidin-TRITC fluorophores (Fig. 3).

### Live Tetracycline staining in differentiating cells

**FIG. 4.**
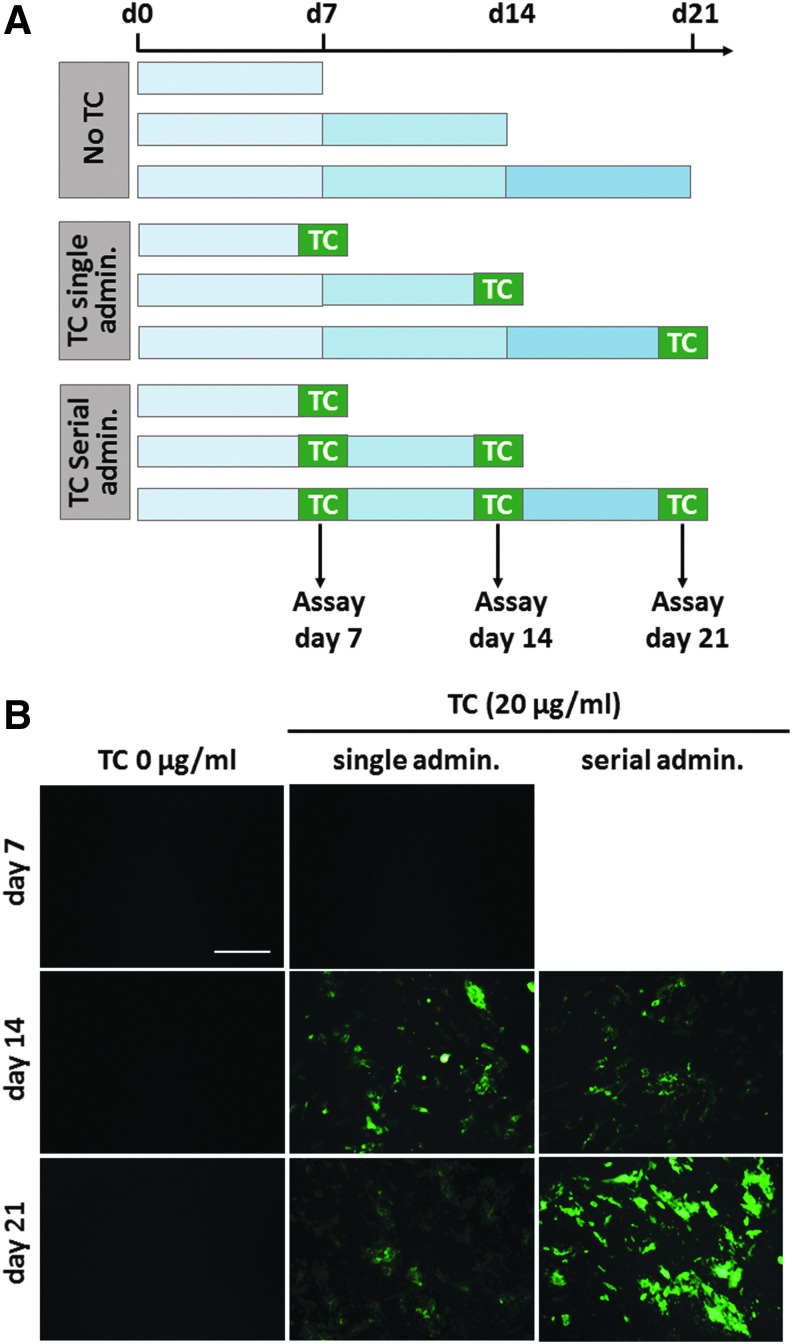
Live fluorescence monitoring of MSC differentiation using single or serial doses of TC. **(A)** Diagram and timeline of TC administration to live cells under osteogenic treatment for 7, 14, and 21 days. **(B)** Representative fluorescence images of OS-treated live MSCs at days 7, 14, and 21 after single and serial TC exposure (20 μg/mL, *green*) compared to unexposed controls. Scale bar: 125 μm. Color images available online at www.liebertpub.com/tec

To further optimize TC staining for live monitoring of osteogenic differentiation, TC administration to live cultures was performed in either single or serial doses (20 μg/mL) at days 6, 13, and 20 (Fig. 4A), to evaluate the biocompatibility and efficiency of multiple TC exposures. Cells exposed to single or repeated TC treatment were first observed under fluorescence microscopy to evaluate the nature and distribution of the staining overtime (Fig. 4B). Live imaging revealed visible TC-stained mineral deposits at days 14 and 21 of OS treatment, but not at day 7, suggesting minimal differentiation at this time point. At day 14, the TC staining pattern and signal intensity were similar in cells exposed to single and serial TC doses. At day 21 of differentiation however, a sharper and more defined signal was observed after serial administration of TC (three consecutive doses) in comparison to a single administration, suggesting a cumulative effect of serial TC treatment from day 14 but not from day 7.

**FIG. 5.**
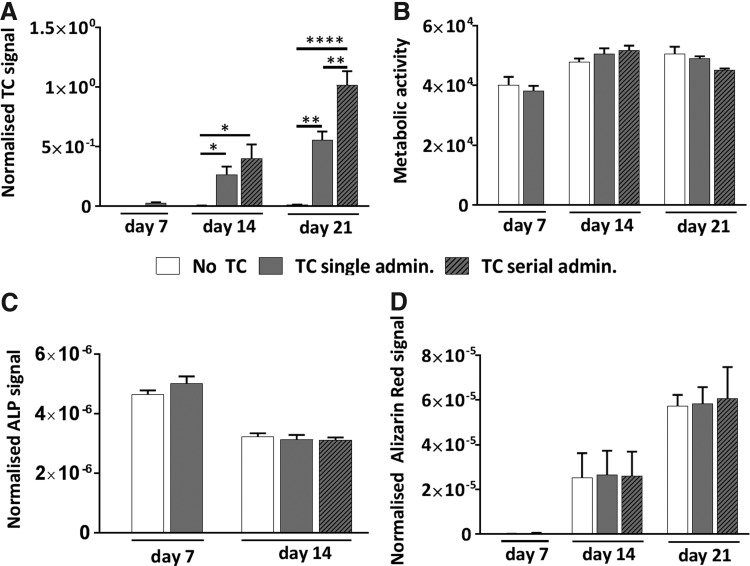
Time course analysis of the single or serial TC exposure (20 μg/mL) on MSC cultures compared to no TC exposure. **(A)** TC fluorescence signal quantitation showing increased signal upon repeated exposure. **(B–D)** Cell metabolic activity **(B)**, ALP activity **(C)**, and Alizarin Red S staining quantitation **(D)** in MSC cultures showing no significant difference across all conditions. *n* = 3. ALP, alkaline phosphatase.

Quantification of TC fluorescence was performed in living cells at days 7, 14, and 21 of OS differentiation to complement the microscopy observation, confirming time-dependent increase in TC signal (Fig. 5A). The administration of serial doses resulted in significantly higher TC fluorescence intensity at day 21 in comparison to the single dose administration protocol. Power analysis performed to evaluate the assay's sensitivity showed that the administration of TC 20 μg/mL had a power of 97% and 100% to discriminate twofold and fivefold differences with three samples in each group, and to discriminate a 1.5-fold difference the assay's power was of 78% with six samples.

When analyzing cellular parameters, TC exposure was not found to negatively affect metabolic activity after either single or serial doses at any of the time points analyzed (Fig. 5B), and measurements of ALP activity at days 7 and 14 showed comparable levels in cells after single or serial TC treatment and cells unexposed to TC (Fig. 5C). The Alizarin Red S staining performed confirmed the progression of cell differentiation from days 7 to 21 (Supplementary Fig. S3). Importantly, staining quantification (Fig. 5D) revealed no significant differences in mineral deposition at any time point between untreated and TC-treated cells, whether using single or serial doses, indicating that TC treatment did not interfere with the progression of cellular differentiation (*p* > 0.9999).

To confirm the versatility of the assay, the serial administration of 20 μg/mL TC was also performed to human primary MSCs, which constitutes a clinical relevant cell type but also to the mouse D1 immortalized MSCs which is widely used in *in vitro* studies (see Supplementary Data). Live fluorescence imaging showed for both cell types a sharp and defined staining pattern at day 21 after three treatments with TC (Supplementary Fig. S4).

## Discussion

The use of *in vitro* live cell assays permits the monitoring and evaluation of cell status in ongoing cultures, allowing longitudinal assessment of cellular responses in real time. In the field of bone research and tissue engineering this is important as *in vitro* studies involve long-term experiments to allow for ECM maturation and mineralization, which are typically considered over 3 weeks of cell culture. The assessment of the ALP enzymatic activity in living cells allows for the evaluation of the status of the cell differentiation in real time during the *in vitro* culture period; however, this targets the early stage of differentiation.^2^ Moreover, molecular reporter systems engineered using osteogenic gene promoters to drive a reporter marker can allow for a direct follow-up of gene expression and differentiation profile^20^; however, these imply genetic manipulations therefore preventing their direct application in primary cells. To overcome these limitations, we have developed a new protocol for the nondestructive measurement of mineralization in live cultures using tetracycline hydrochloride. We have also defined the optimal regime of TC administration to enable staining, quantification, and fluorescence imaging in live cells using human MSCs as a differentiation model. We have also showed that TC staining can be multiplexes with other fluorophores to enable advanced cellular analysis of osteogenic cultures.

Among the several tetracycline derivatives available and already in use for *in vivo* bone labeling, we selected tetracycline hydrochloride as it displayed the highest brightness in comparison to others when used to label rat bone samples.^21^ Our results showed that TC administrated *in vitro* as single or repeated doses did not alter the metabolic activity of human MSCs. The results of several studies suggest that different administration protocols and/or TC derivatives may differently affect cell health and also that sensitivity to TC derivatives might be cell type dependent. Indeed, MG-63 human osteosarcoma cells experienced a decrease in number after overnight incubation with 10 μg/mL doxycycline and a significant reduction of proliferation after daily treatment with the same dose of the compound.^22^ The treatment of primary human osteoblasts with 190 μg/mL of TC resulted in a 30% increase of lactate dehydrogenase, which was considered as an indication of impaired mitochondrial function, and suggested that this cytotoxic effect could be partially mediated by an alteration of mitochondrial respiration.^23^ Another study reported that the administration of TC analogs (doxycycline, COL-3, and minocycline) from 10 μg/mL to the acute myeloid leukemia cell line HL60 reduced cell viability of >50%. By contrast, 1 μg/mL of doxycycline and minocycline continuously added to the cell culture medium have been reported to significantly increase the proliferation of human osteoblastic bone marrow cells.^24,25^ Our results also showed that TC administration at days 7 and 14 during the culture period did not interfere with the induction and progression of the differentiation process. For both ALP activity and mineral deposition assays, no significant differences were observed between cells treated with single and serial doses of TC in comparison to cultures unexposed to TC. In this regard too, existing reports on the effect of TC treatment on bone differentiation are contradictory and might depend on the TC derivative used, the dose, and the administration regime. *In vitro*, the daily administration of 1 μg/mL of doxycycline or minocycline seemed to significantly promote the mineralization of human bone marrow-derived osteoblasts.^25^ *In vivo*, several studies performed on diverse disease models (such as diabetes, osteopenia, and osteoporosis) in mice and rats reported a positive association between the administration of TC derivatives and bone formation and density.^26–29^ However, it has been proposed that such an effect observed *in vivo* might be mediated by an inhibitory effect of TC on osteoclast function.^29,30^ As an opposite trend, TC administration at early stages of development or to *ex vivo* embryonic bones, bone growth was compromised followed by increase in bone fragility.^31,32^

The present study also demonstrated that TC is a suitable compound for the evaluation of osteogenic differentiation in living cells such as MSCs, over an extended culture period. All the TC doses tested in this study (5–40 μg/mL) were selected within the same range as previously reported^14,32^ and were suitable for the fluorescent labeling of mineralizing cells, resulting in a more defined signal as the concentration increased. This trend was confirmed by spectrophotometric analysis, which revealed a linear increase of the detected fluorescence signal. However, among the TC doses tested in this study, 20 μg/mL was selected as optimum to monitor the progression of MSC differentiation at various time points. While the highest dose (40 μg/mL) provided a more defined and brighter signal in fluorescent images, the spectrophotometric quantitation also showed a significant increase of the signal in undifferentiated cells, suggesting the increase of unspecific background signal at this concentration. In our study, TC labeling was clearly visible as green stain using a conventional DAPI long pass filter at days 14 and 21, but not at day 7 of osteogenic treatment. This result was in agreement with the spectrophotometric quantitation of TC fluorescence signal and was also confirmed by parallel Alizarin Red S staining, which did not reveal any detectable mineral deposits at day 7. Due to the calcium tropism of TC, the presence of calcium phosphate minerals in the ECM is necessary for the staining. The production of mineralized ECM is a late event during bone-lineage differentiation, starting around days 10–12, while the earlier period is characterized by active cell proliferation and production of ECM components.^2^ Serial TC administration resulted in a stronger and more defined staining in MSC cultures, particularly at day 21, in comparison to the single dose administration. This result, observed by microscopy and confirmed by spectrophotometry, suggests the retention and accumulation of the dye into the mineralizing bone-like nodules between the different administrations over the 21 days of culture. This appears in agreement with *in vivo* studies reporting the retention and visualization of this compound in bones up to 4 weeks after administration.^33^ These results confirm the suitability of TC staining to monitor the progression of live MSC differentiation at various time points, using a simple and cell neutral protocol.

The real-time evaluation of mineralizing cultures has previously been achieved by the continuous administration of Calcein without affecting the cell viability or the progression of differentiation, resulting in a green labeling visible through a FITC long pass filter.^8^ However, this method is based on the uptake of Calcein by living cells; therefore its use is limited to live cultures while the TC can also be used in fixed samples.^3,13^ In this regard, it is worth noting that TC allows for the multiplex staining with fluorophores visible in other channels, including the nuclear dye Hoechst 33258 and TRITC-conjugated Phalloidin as shown in our study, and is therefore a versatile tool for immunohistochemistry analysis. Moreover, our results show that the quantitative assessment of mineralization at specific time points can be achieved by an incubation of <24 h with TC resulting in quantifiable and visible signal.

In summary, in this study we have developed a new protocol for the quantitative monitoring and quantitation of cell mineralization in real time based on the use of tetracycline hydrochloride. In comparison to conventional histological methods such as Alizarin Red S and Von Kossa staining, which are end point assays and require multiple steps for staining and dye quantification, the use of TC is cytocompatible, can be performed in live cells lowering the number of cells required for longitudinal studies, and is both straightforward and economical. Overnight incubation results in visible and quantifiable fluorescence signal, which can be quantified by spectrophotometry directly from live cultures while being compatible with the use of other fluorophores in live and fixed samples, enabling multiplex immunohistochemistry analysis. TC cell labeling enables the *in vitro* evaluation of osteogenic differentiation in different cell models, including human primary stem cells, and can thus support research into bone repair and tissue engineering targeting new pro-osteogenic approaches.

## Conclusions

In this study, we have described a new nondestructive method for the live and quantitative staining of MSC cultures undergoing osteogenic differentiation, based on the transient administration of tetracycline hydrochloride during the culture period. The results showed this method to be nontoxic, sensitive, and quantitative. TC can be selectively imaged by fluorescent microscopy in live cells and enables the concomitant use of other fluorophores, which offers a useful and versatile method for high throughput analysis of osteogenic cultures.

## Supplementary Material

Supplemental data
