# Supplementary material for: Live Quantitative Monitoring of Mineral Deposition in Stem Cells Using Tetracycline Hydrochloride
Source: Tissue Eng Part C Methods. 2018 Mar 1;24(3):171–8. doi: 10.1089/ten.tec.2017.0400 (PMC5865259; doi:10.1089/ten.tec.2017.0400)
Supplement: Supplemental data [file Supp_Data.pdf]

Supplementary Data

Supplementary Methods

Primary (Lonza, UK) and mouse (ATCC CRL-12424) bone marrow-derived mesenchymal stem cells were seeded in 48-well plates at a density of 4,000 cells/cm^2 and 50,000 cells/cm^2, respectively, and treated as described in the main text.

For DNA amount semiquantitation, cells were seeded at a density of 20,000 cells/cm^2 in 48-well plate, and PicoGreen® dsDNA quantitation reagent was used according to the manufacturer's guidelines. Briefly, samples were washed twice with phosphate buffered saline, and 100 μL of sterile distilled water was added to each one; samples were then frozen and thawed thrice, and 95 μL was transferred to a 96-well plate. 95 μL of a 1× solution of PicoGreen dsDNA quantitation reagent diluted in TRIS-EDTA buffer 1× was added to each sample and incubated in the dark during 5 min. Measurement of fluorescence was performed in a microplate reader using 480 and 520 nm as excitation and emission wavelengths, respectively.

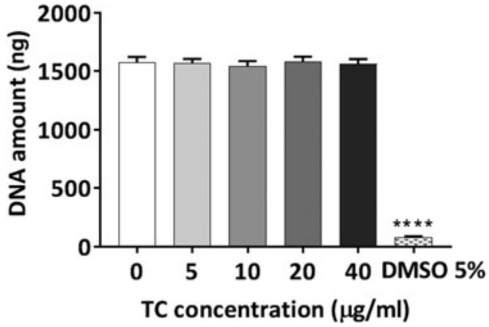

SUPPLEMENTARY FIG. S1. Semiquantitation of DNA amount in human MSCs exposed to increasing doses of TC at day 2. Cells treated with DMSO 5% were included as cytotoxic control. ****$p < 0.0001$ ($n = 5$). DMSO, dimethyl sulfoxide; MSCs, mesenchymal stem cells.

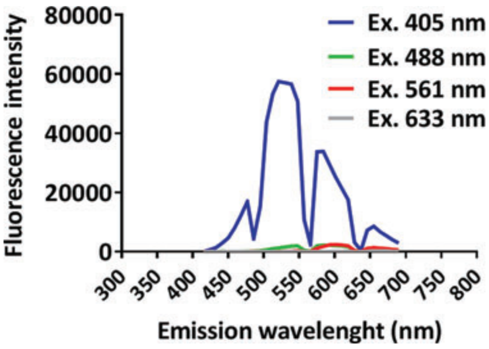

SUPPLEMENTARY FIG. S2. Full emission spectrum of TC using 405, 488, 561, and 633 nm excitation wavelengths.

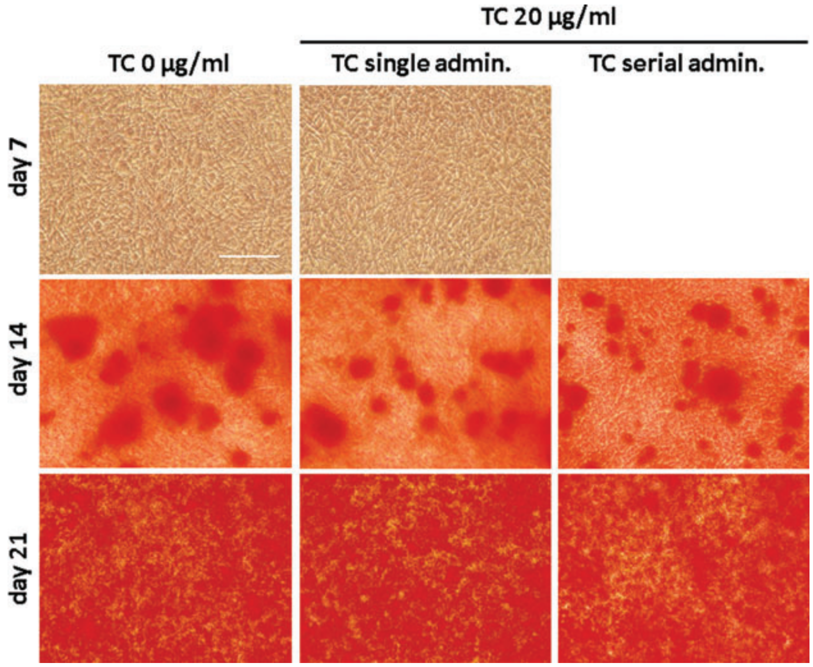

SUPPLEMENTARY FIG. S3. Representative images of Alizarin Red S staining performed on MSC cultures treated with single or serial TC exposure (20 μg/mL) compared to no-TC exposure.

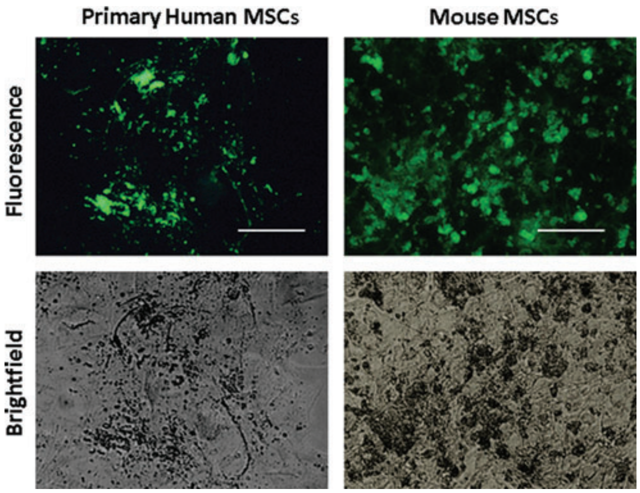

SUPPLEMENTARY FIG. S4. Representative live images taken in OS-treated primary human MSCs and mouse MSCs after serial TC exposure (20 μg/mL, *green*) at 20 and 13 days, respectively. Scale bar: 125 μm. OS, osteogenic medium.
